# Orthogonal Experimental Research on Dielectrophoresis Polishing (DEPP) of Silicon Wafer

**DOI:** 10.3390/mi11060544

**Published:** 2020-05-27

**Authors:** Tianchen Zhao, Qianfa Deng, Cheng Zhang, Kaiping Feng, Zhaozhong Zhou, Julong Yuan

**Affiliations:** 1 Key Laboratory of Air-driven Equipment Technology of Zhejiang Province, Quzhou University, Quzhou 324000, China; fengkaiping11@126.com (K.F.); zzz_2227@163.com (Z.Z.); 2 Xinchang Research Institute of ZJUT, Zhejiang University of Technology, Xinchang 312500, China; 3 School of Mechanical Engineering, Shandong University, Jinan 250000, China; 4 Ultra-precision Machining Centre, Zhejiang University of Technology, Hangzhou 310014, China; jlyuanzjut@163.com; 5 College of Mechanical and Electrical Engineering, Wenzhou University, Wenzhou 325035, China; zhangcheng@wzu.edu.cn

**Keywords:** silicon wafer, dielectrophoresis polishing, orthogonal experiment, material removal rate, surface roughness

## Abstract

Silicon wafer with high surface quality is widely used as substrate materials in the fields of micromachines and microelectronics, so a high-efficiency and high-quality polishing method is urgently needed to meet its large demand. In this paper, a dielectrophoresis polishing (DEPP) method was proposed, which applied a non-uniform electric field to the polishing area to slow down the throw-out effect of centrifugal force, thereby achieving high-efficiency and high-quality polishing of silicon wafers. The principle of DEPP was described. Orthogonal experiments on important polishing process parameters were carried out. Contrast polishing experiments of silicon wafer were conducted. The orthogonal experimental results showed that the influence ratio of electric field intensity and rotation speed on material removal rate (MRR) and surface roughness was more than 80%. The optimal combination of process parameters was electric field intensity 450 V/mm, rotation speed 90 rpm, abrasive concentration 30 wt%, size of abrasive particle 80 nm. Contrast polishing experiments indicated that the MRR and material removal uniformity of DEPP were significantly better than traditional chemical mechanical polishing (CMP). Compared with the traditional CMP, the MRR of DEPP was increased by 17.6%, and the final surface roughness of silicon wafer reached *R*a 0.31 nm. DEPP can achieve high-efficiency and high-quality processing of silicon wafer.

## 1. Introduction

Silicon has become the most mainstream basic material in micromachines, microelectronics, and microelectromechanical systems (MEMS) because of its unique physical and chemical properties and rich mineral resources (about 26% of the earth’s crust weight, second only to oxygen) [1,2,3,4]. At the macro scale, when the relative roughness is less than 5%, the effect of roughness is often neglected. However, in micromachines and MEMS, due to the micro-nano size, the ratio of surface area to volume increases significantly, so that the surface effect increases [5,6,7,8,9], and the effect of surface quality becomes very prominent. For example, Broer found that poor surface quality has a detrimental effect on the availability of stable equilibria of MEMS [10]. Pandey found that the surface roughness will increase the damping of the squeeze film in the MEMS structure [11]. Li found that surface roughness also affects the contact performance of MEMS switches [12]. In addition, some researchers found that surface quality has an effect on the electromechanical performance of RF-MEMS capacitive switches, and the poor surface quality reduces down-state capacitance of RF-MEMS capacitors [13,14]. Therefore, excellent surface quality is very important for silicon wafer used in micromachines.

At present, most silicon wafer and corresponding micromachines use polishing as their final processing [15,16]. Chemical mechanical polishing (CMP) is one of the most widely used processing methods to obtain high surface quality in batch production [17,18]. The principle is that the polishing slurry is injected into the polishing area, and the workpiece material is removed through the relative rotational contact of the polishing plate and the workpiece surface [19]. Due to the rotation of the polishing plate, the polishing slurry quickly leaves the polishing area under the action of centrifugal force, which results in a lower polishing efficiency; At the same time, the polishing slurry is unevenly distributed by the centrifugal force, and the surface quality of polishing cannot be further improved [20,21].

To solve the above-mentioned problems of CMP, a dielectrophoresis polishing (DEPP) method based on the dielectrophoretic effect was proposed. It has been proved that the DEPP is feasible to restrain the problems caused by centrifugal force [22,23,24]. In order to achieve a high efficiency and high surface quality polishing method of DEPP, the influence of process parameters on the DEPP results needs to be further researched. In this paper, silicon wafers were taken as the research object, and the optimal process parameters for silicon wafer polishing were obtained through orthogonal experiments of DEPP. Based on the optimized combination of process parameter, a silicon wafer contrast polishing experiment with the traditional CMP was carried out.

## 2. Dielectrophoresis Polishing Method

**Figure 1:**
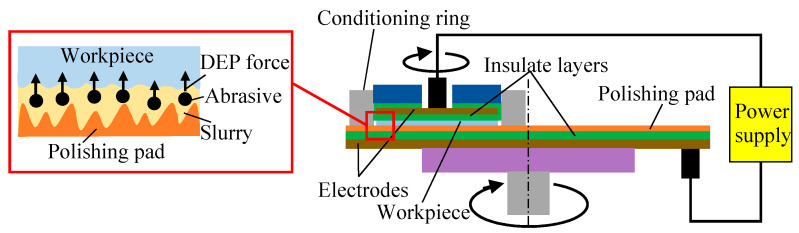
Mechanism and apparatus of DEPP.

**Figure 2:**
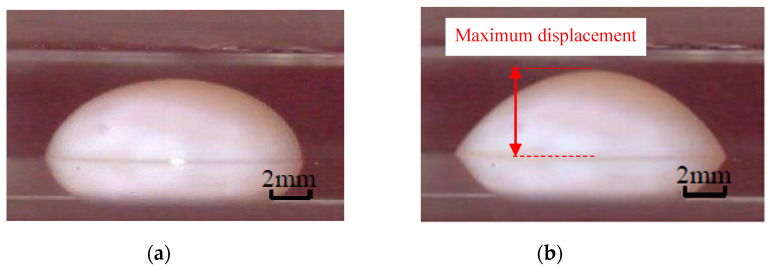
Displacement of the polishing slurry in non-uniform electric field: (**a**) Initial state; (**b**) Maximum displacement.

Dielectrophoresis (DEP) is the translational motion of electrically neutral particles caused by their polarization effects in a non-uniform electric field [25,26,27]. DEPP utilizes the DEP effect of abrasives in a non-uniform electric field to assist polishing. The mechanism and apparatus of DEPP are shown in Figure 1. DEPP method applies a non-uniform electric field in the vertical direction of the traditional CMP processing area, and the abrasives in polishing slurry between the polishing pad and the workpiece are polarized. The polarized abrasives move toward the surface of the workpiece under the action of DEP force, which increases the friction with the surface of the workpiece, thereby slowing down the throw-out effect of centrifugal force. Therefore, the residence time of the polishing slurry in the processing area is extended, the distribution uniformity of the polishing slurry in the processing area is improved, the polishing efficiency and quality are improved, and high-efficiency and high-quality polishing is realized. Figure 2 shows the upward displacement of the polishing slurry by DEP force in a non-uniform electric field [28].

As a novel polishing method, DEPP needs to further research the impact of various process parameters on polishing results, and through systematic polishing experiments to determine and optimize DEPP process parameters.

## 3. Experimental Design

Orthogonal experimentation is a high-efficiency, fast, and economical experimental design method, which can analyze the experimental results through fewer experiments and obtain better process parameters [29,30,31]. In order to obtain high-efficiency and high-quality silicon wafer polishing, surface roughness (*R*a) and material removal rate (MRR) are usually selected as evaluation indicators. Therefore, the effects of process parameters on the surface roughness and MRR of silicon wafer polishing were researched through orthogonal experiments.

There are many factors that affect the polishing results of DEPP. It is very important for DEPP to select the reasonable process parameters. Based on the principle and mechanism of DEPP, the most important process parameters that affect the surface roughness and MRR of DEPP are electric field intensity, abrasive concentration, size of abrasive particle, and rotation speed of polishing plate.

According to the number of process parameters, a 4-factor, 3-level L9 (3^4^) standard orthogonal table was selected for polishing experiment. Due to the limited adjustable range of the special high-voltage power supply, the electric field intensity was set to 150, 300, and 450 V/mm. According to the actual production demand and performance of polishing apparatus, the rotation speeds of the polishing plate and workpiece were selected to be 30, 60, and 90 rpm. Combined with the actual processing conditions, the size of the SiO_2_ abrasive particles were 30, 80, and 110 nm, and the abrasive concentrations were 10, 20, and 30 wt%. The four factors and three levels in the polishing experiment are shown in Table 1.

Fill the above factors and levels into the L9 (3^4^) orthogonal table, as shown in Table 2. The last two columns in the table are the MRR and the final surface roughness, respectively, which are the indexes examined of the experiment. Each row in the table represents a combination of parameters for an experiment. All four columns are balanced, orthogonal, and independent. A total of nine groups of DEPP experiments with different combination of process parameter need to be conducted.

**Figure 3:**
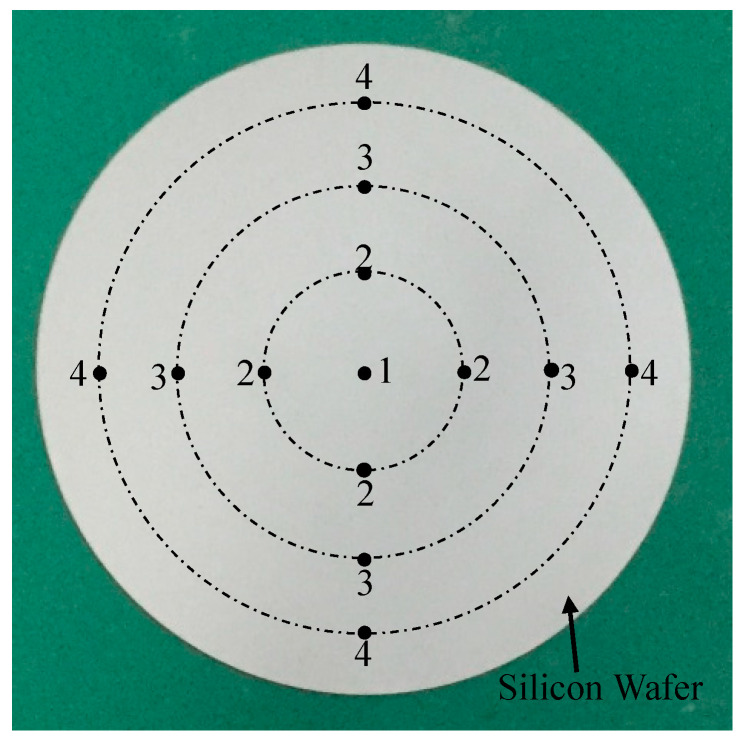
Measurement points in silicon wafer.

The polishing object was 3-inch silicon wafer, the process before polishing was lapping, and the surface roughness of the silicon wafer before polishing was about 400 nm. In order to research the influence of process parameters on the falling speed of surface roughness, the surface roughness of silicon wafer was measured after 2 h of polishing. The measurement points are shown in Figure 3. Point 1 is at the center of the circle, points 2, 3, and 4 are three points at different diameters (20, 40, and 60 mm). A total of 13 points need to be measured on each silicon wafer surface. The average value of all the surface roughness (13 points) is the surface roughness value in the Table 2. The MRR was calculated according to the quality change before and after polishing. The experiment results are show in the last two columns of Table 2. 

## 4. Results and Discussion

### 4.1. Experimental Data Analysis Method

The signal to noise ratio (S/N) represents the degree of interference by noise factors, unit is dB. The S/N was used as the evaluation feature in the optimization design and analysis of orthogonal experimental results. When the evaluation object is the MRR of silicon wafer, it has a ‘the-larger-the-better’ characteristic, and the calculation equation of S/N is (1). When the evaluation object is the surface roughness of silicon wafer, it has a ‘the-smaller-the-better’ characteristic, and the equation of S/N is (2). When using S/N to analyze the average response of each process parameter in the orthogonal experiment, the larger the S/N, the better the result.

(1) $$S/N_{i}=-10\log\frac{1}{w}\overset{r}{\underset{j=1}{\sum }}\frac{1}{H_{ij}^{2}}$$

(2) $$S/N_{i}=-10\;\log\;\frac{1}{w}\overset{r}{\underset{j=1}{\sum }}R_{ij}^{2}$$

*i* is the experiment number, *w* is the number of different detection points, *H_ij_* and *R_ij_* are respectively the measurement values of MRR and surface roughness in the No. *i* experiment.

Analysis of variance (ANOVA) was used to evaluate the influence ratio of the response of each process parameter on the results. ANOVA usually uses the sum of the squares of the standard deviation to calculate and analyze the difference and the degree of difference of each factor. Its basic characteristic is that the total corrected of sum of squares *SS_T_* is equal to the sum of squares of treatment *SS_K_* and the sum of squares of error *SS_E_*.

Since the error column was not set in this experiment, the total corrected of sum of squares *SS_T_* can be expressed as

*SS_T_* = *SS_A_* + *SS_B_* + *SS_C_* + *SS_D_* (3)

At the same time, the total corrected of sum of squares *SS_T_* can also be expressed as

(4) $$SS_{T}=\overset{n}{\underset{i=1}{\sum }}{\left(y_{i}-\bar{y}\right)}^{2}=\overset{n}{\underset{i=1}{\sum }}y_{i}^{2}-2n{\bar{y}}^{2}+n{\bar{y}}^{2}=\overset{n}{\underset{i=1}{\sum }}y_{i}^{2}-\frac{M^{2}}{n}$$

*y_i_* is the S/N of the No. *i* experimental result, i.e., S/N*_i_*. $M=\sum_{i=1}^{n}y_{i}^{2}$ is the sum of the S/N of all experimental results, *n* is the total number of experiments.

The sum of squares of treatment *SS_K_* is

(5) $$SS_{k}=\overset{t}{\underset{j=1}{\sum }}tx{\left({\bar{y}}_{j}-\bar{y}\right)}^{2}=\overset{t}{\underset{j=1}{\sum }}\left(\frac{Sy_{j}^{2}}{t}\right)-\frac{M^{2}}{n}$$

*k* represents a factor, i.e., A (electric field intensity), B (rotation speed), C (abrasive concentration), and D (size of abrasive particle). *j* is the level number of factor *k*. ${\bar{y}}_{j}$ is the average value of each level of factor *k*. *t* is the number of repetitions at each level of a factor, here *t* is 3. *Sy_j_* is the sum of all *y_j_* under each factor *k*.

### 4.2. S/N Average Response Analysis

**Figure 4:**
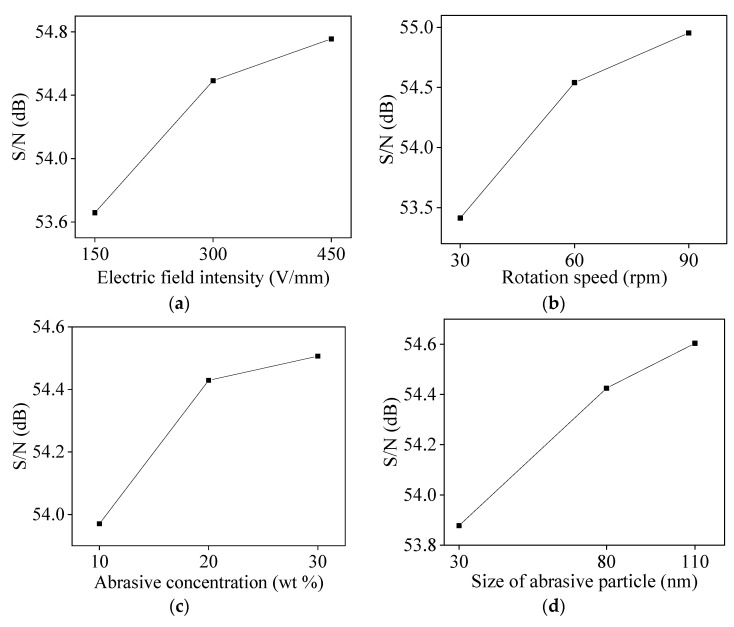
Influence of process parameter level on material removal rate (S/N average response): (**a**) electric field intensity; (**b**) rotation speed; (**c**) abrasive concentration; (**d**) size of abrasive particle.

**Figure 5:**
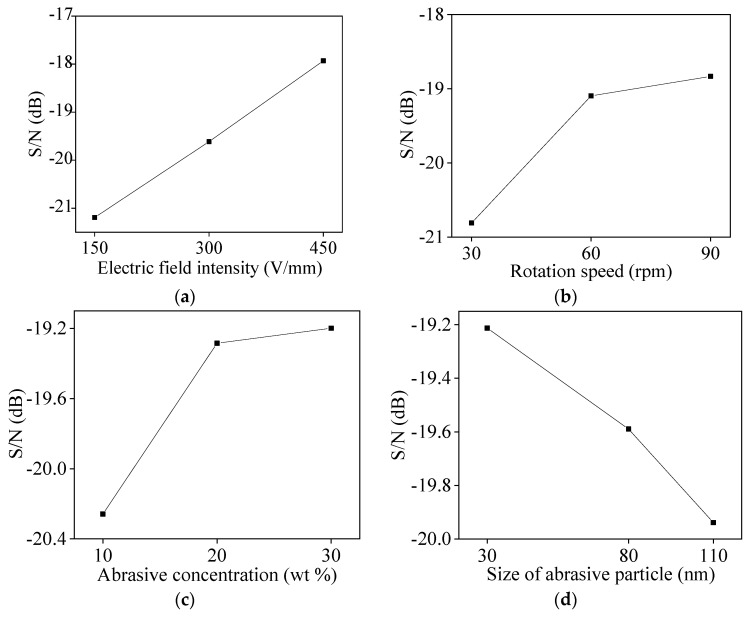
Influence of process parameter level on surface roughness (S/N average response): (**a**) electric field intensity; (**b**) rotation speed; (**c**) abrasive concentration; (**d**) size of abrasive particle.

The experimental data were substituted into Equations (1) and (2) to obtain the average values of S/N of the four factors (process parameters) as shown in Table 3, Table 4, Table 5 and Table 6. Figure 4 and Figure 5 are respectively the influence of levels of process parameter on MRR and surface roughness based on average value of S/N.

The larger the S/N, the higher the MRR of silicon wafer. As shown in Figure 4, the MRR increases with the increase of electric field intensity, rotation speed of polishing plate, abrasive concentration, and size of abrasive particle. This is because the greater the electric field intensity, the greater the DEP force of the abrasive particles, so the greater the removal effect of the abrasive on the surface of the silicon wafer. The greater the rotation speed of the polishing plate, the greater the relative speed between the abrasive and the silicon wafer, so the greater the MRR. As the abrasive concentration increases, the number of abrasive participating in the polishing increases, so the MRR increases. However, due to the limitation of the contact area, after the number of abrasives participating in the material removal reaches a certain value, it may come close to the saturation state. At this time, if the abrasive concentration continues increasing, the increase in the MRR is small. The larger the size of the abrasive particle, the deeper the removal effect, thereby the MRR is improved.

The larger the S/N, the smaller the surface roughness of silicon wafer. As shown in Figure 5, the surface roughness decreases with the increase of electric field intensity, rotation speed of polishing plate, and abrasive concentration, and increases with the increased of size of abrasive particle. The reason is that the greater the electric field strength, the greater the DEP force of abrasive. Therefore, when the more abrasives remove the micro-peaks on the surface of the silicon wafer, the smoother surface and the smaller surface roughness are obtained; The larger the rotation speed of the polishing plate, the greater the relative speed of the abrasive and the silicon wafer, and the DEP force can effectively slow the effect of the abrasive being thrown away by the centrifugal force. With more abrasives participating in the material removal, the MRR becomes higher, and the surface roughness of the silicon wafer decreases. The larger the abrasive concentration, the more abrasives actually participate in material removal, and the lower the surface roughness is. Similarly, when the abrasive is close to saturation, if the abrasive concentration still increases, the surface roughness will decrease slowly. The larger the size of abrasive particle, the greater the depth of material removal on the surface of silicon wafer, so the surface roughness increases with the increase of size of abrasive particle.

According to the analysis results of average response of S/N, for the MRR, the optimal combination of process parameters is electric field intensity 450 V/mm, rotation 90 rpm, abrasive concentration 30 wt%, size of abrasive particle 110 nm. For surface roughness, the best combination of process parameters is electric field intensity 450 V/mm, rotation speed 90 rpm, concentration abrasive 30 wt%, size of abrasive particle 30 nm.

### 4.3. Analysis of Variance

ANOVA was used to analyze the influence ratio of the four factors (process parameters) on the MRR and surface roughness. According to Equations (3)–(5), the variance of MRR and surface roughness was calculated. The influence ratio of electric field intensity, rotation speed, abrasive concentration and size of abrasive particle on MRR, and surface roughness are shown in Table 7 and Table 8.

For the MRR, the order of influence ratio of process parameters is rotation speed > electric field intensity > size of abrasive particle > abrasive concentration. For surface roughness, the order of influence ratio of process parameters is electric field intensity > rotation speed > abrasive concentration > size of abrasive particle.

It can be seen from Table 7 and Table 8 that the influence ratio of electric field intensity and rotation speed on MRR and surface roughness is more than 80%, which are the most important influence factors. Increasing the electric field intensity can increase the MRR and reduce the surface roughness. The greater the electric field intensity, the stronger the ability of the DEP force to slow down the abrasive being thrown out of the polishing area by centrifugal force. Therefore, the rotation speed of polishing plate can be increased appropriately to continue improving the MRR. At the same time, it guarantees the polishing quality, which reflects the advanced nature of the DEPP method.

According to the ANOVA, the influence ratio of the size of abrasive particle on the MRR and surface roughness is relatively small. However, in actual polishing, it is necessary to consider both the MRR and the surface roughness, so the size of abrasive particle 80 nm should be selected as the optimal level after comprehensive consideration.

As mentioned above, the optimal combination of process parameters is electric field intensity 450 V/mm, rotation speed 90 rpm, abrasive concentration 30 wt%, size of abrasive particle 80 nm.

## 5. Contrast Experiment of Polishing

In order to verify that DEPP is a high-efficiency and high-quality polishing method, on the basis of the above orthogonal experiment results, the optimized combination of process parameters was used to carry out a contrast experiment with traditional CMP method. The polishing object was a 3-inch silicon wafer. The conditions of the two sets of polishing contrast experiments were almost identical. The only difference was whether there was a non-uniform electric field in the polishing processing area. Turn off the special high-voltage power supply on the DEPP apparatus can realize the traditional CMP method. The detailed polishing conditions are shown in Table 9. The surface roughness was measured at different points on the center of the wafer—diameters of 20, 40, and 60 mm—every 30 min. By comparing the changes of surface roughness in different diameters, the polishing uniformity of the two polishing methods was analyzed. The quality change of silicon wafer in the same time was measured and the MRR of the two methods was compared.

**Figure 6:**
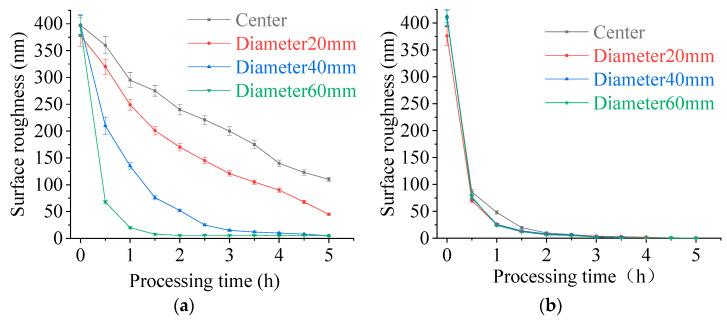
Variation of surface roughness at different diameters of silicon wafer: (**a**) traditional CMP; (**b**) DEPP.

Figure 6a is the variation of surface roughness of silicon wafer at different diameters using traditional CMP. It can be seen from the figure that the surface roughness at different diameters decreased unevenly, and the decrease rate becomes slower as the diameter decreases. Figure 6b is the variation of surface roughness of silicon wafer at different diameters processing by DEPP. From the figure, it can be seen that the surface roughness of each diameter of silicon wafer decreases uniformly, only the center of the silicon wafer decreases slightly slower than other diameters.

**Figure 7:**
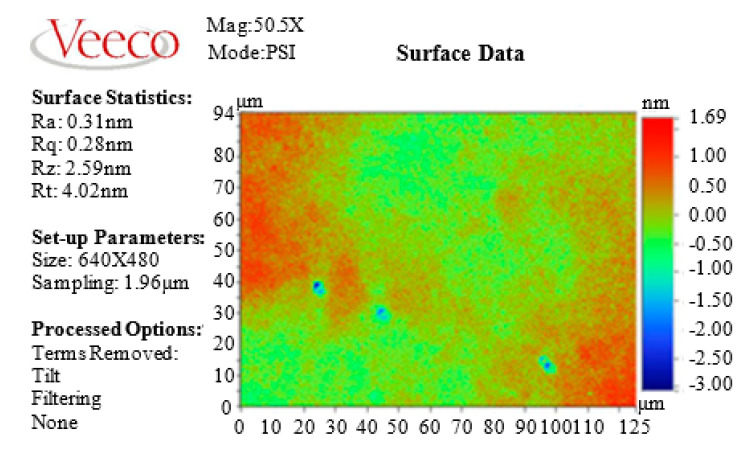
Final surface roughness of silicon wafer processed by DEPP.

**Figure 8:**
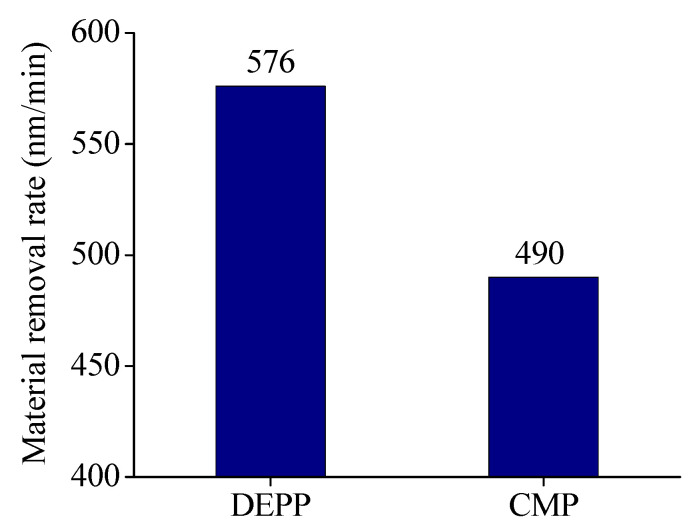
Comparison of MRR between two polishing methods.

Comparing Figure 6a,b, it is found that the two polishing methods have completely different decrease rates of surface roughness at the same diameter. At the center of the silicon wafer, diameter of 20 mm, and diameter of 40 mm, the surface roughness of the silicon wafer polished with DEPP decreased much faster than that of traditional CMP. The surface roughness of each diameter is less than 10 nm after polishing by DEPP for 2 h. While using traditional CMP to polish silicon wafer, only the surface roughness at diameter of 60 mm is less than 10 nm after 2 h. The diameter of 40 mm, the diameter of 20 mm, and the center of silicon wafer need to be polished for 4.5 h, 7 h, and 8 h respectively, for the surface roughness to be less than 10 nm. After polishing by DEPP for 3 h, the final surface roughness of the silicon wafer reached *R*a 0.31 nm. Figure 7 is the final surface roughness and morphology of the silicon wafer measured using a Veeco white light interferometer. The optimized process parameters were used, and the polishing time of DEPP was 3 h, so the surface roughness is much smaller than that in orthogonal experiments. Figure 8 shows the MRR of silicon wafers polishing by DEPP and traditional CMP. The MRR of DEPP is 576 nm/min, which is an increase of 17.6% compared to 490 nm/min of traditional CMP. Using optimized process parameters for silicon wafer polishing, the polishing results (surface roughness and MRR) are also improved compared with the previous research work [19].

The above experimental results come from abrasives in polishing slurry subjected to the DEP force which were then moved to the silicon wafer surface, which not only increases the distribution density of the abrasive on the silicon wafer surface, but also increases the friction force between the abrasive and the silicon wafer surface. The DEP force restrains the throw-out effect of centrifugal force, which can prolong the residence time of polishing slurry and improve the polishing slurry distribution uniformity in the polishing area.

Therefore, compared with the traditional CMP, the DEPP method has better performance for polishing silicon wafer, the surface roughness decreases faster, and the material removal is more uniform and faster.

## 6. Conclusions

In order to obtain the optimal process parameters combination of DEPP and achieve high-efficiency and high-quality polishing of silicon wafer, an orthogonal experiment of DEPP of silicon wafers was carried out. According to the optimized combination of process parameters, a silicon wafer contrast polishing experiment was conducted.

The electric field intensity, abrasive concentration, size of abrasive particle, and rotation speed of polishing plate—which are the most important factors affecting on the surface roughness and MRR—were selected as the process parameters. A four-factor, three-level L9 (3^4^) standard orthogonal experiment was carried out. The analysis of S/N average response shows that the MRR increases with the increase of electric field intensity, rotation speed of polishing plate, abrasive concentration, and size of abrasive particle. The surface roughness decreases with the increase of electric field intensity, rotation speed of polishing plate, and abrasive concentration, and increases with the increase of size of abrasive particle. The ANOVA indicates that the influence ratio of electric field intensity and rotation speed on MRR and surface roughness is more than 80%, which are the most important influence factors. The optimal combination of process parameters is electric field intensity 450 V/mm, rotation speed 90 rpm, abrasive concentration 30 wt%, and size of abrasive particle 80 nm.

Contrast polishing experiments show that the MRR and material removal uniformity of DEPP are significantly better than traditional CMP. Compared with the traditional CMP, the MRR of DEPP is increased by 17.6%, and the final surface roughness of silicon wafer reaches *R*a 0.31 nm. DEPP can achieve high-efficiency and high-quality processing of silicon wafers.

## Figures and Tables

**Table 1:** Factors and levels of DEPP process.

A: Electric Field Intensity (V/mm)	B: Rotation Speed (rpm)	C: Abrasive Concentration (wt%)	D: Size of Abrasive Particle (nm)
150	30	10	30
300	60	20	80
450	90	30	110

**Table 2:** L9 (3^4^) table of DEPP process parameter.

Experiment No.	Process Parameter				Experiment Results	
	A: Electric Field Intensity (V/mm)	B: Rotation Speed (rpm)	C: Abrasive Concentration (wt%)	D: Size of Abrasive Particle (nm)	MRR (nm/min)	*R*a (nm)
1A	150	30	10	30	398.7	13.7
2B	150	60	20	80	509.7	10.5
3C	150	90	30	110	550.5	10.5
4D	300	30	20	110	503	11.1
5E	300	60	30	30	531.5	8.3
6F	300	90	10	80	558.1	9.5
7G	450	30	30	80	512.5	8.7
8H	450	60	10	110	560	8.4
9I	450	90	20	30	569.5	6.7

**Table 3:** Average value of S/N of electric field intensity.

A: Electric Field Intensity (V/mm)	Experiment No.	S/N (dB)		Average Value of S/N (dB)	
		MRR	*R*a	MRR	*R*a
150	1A	52.01	−22.7344	53.66	−21.19
	2B	54.15	−20.4238		
	3C	54.82	−20.4238		
300	4D	54.03	−20.9065	54.49	−19.61
	5E	54.51	−18.3816		
	6F	54.93	−19.5545		
450	7G	54.19	−18.7904	54.76	−17.93
	8H	54.96	−18.4856		
	9I	55.11	−16.5215		

**Table 4:** Average value of S/N of rotation speed.

B: Rotation Speed (rpm)	Experiment No.	S/N (dB)		Average Value of S/N (dB)	
		MRR	*R*a	MRR	*R*a
30	1A	52.01	−22.7344	53.41	−20.81
	4D	54.03	−20.9065		
	7G	54.19	−18.7904		
60	2B	54.15	−20.4238	54.54	−19.10
	5E	54.51	−18.3816		
	8H	54.96	−18.4856		
90	3C	54.82	−20.4238	54.95	−18.83
	6F	54.93	−19.5545		
	9I	55.11	−16.5215		

**Table 5:** Average value of S/N of abrasive concentration.

C: Abrasive Concentration (wt %)	Experiment No.	S/N (dB)		Average Value of S/N (dB)	
		MRR	*R*a	MRR	*R*a
10	1A	52.01	−22.7344	53.97	−20.26
	6F	54.93	−19.5545		
	8H	54.96	−18.4856		
20	2B	54.15	−20.4238	54.43	−19.28
	4D	54.03	−20.9065		
	9I	55.11	−16.5215		
30	3C	54.82	−20.4238	54.51	−19.20
	5E	54.51	−18.3816		
	7G	54.19	−18.7904		

**Table 6:** Average value of S/N of size of abrasive particle.

D: Size of Abrasive Particle (nm)	Experiment No.	S/N (dB)		Average Value of S/N (dB)	
		MRR	*R*a	MRR	*R*a
30	1A	52.01	−22.7344	53.88	−19.21
	5E	54.51	−18.3816		
	9I	55.11	−16.5215		
80	2B	54.15	−20.4238	54.42	−19.59
	6F	54.93	−19.5545		
	7G	54.19	−18.7904		
110	3C	54.82	−20.4238	54.60	−19.94
	4D	54.03	−20.9065		
	8H	54.96	−18.4856		

**Table 7:** ANOVA of MRR.

Factors	DOF	SS	SS%
Electric field intensity	2	1.97	27.6
Rotation speed	2	3.81	53.4
Abrasive concentration	2	0.50	7
Size of abrasive particle	2	0.86	12
Total	8	7.14	100

**Table 8:** ANOVA of surface roughness.

Factors	DOF	SS	SS%
Electric field intensity	2	15.96	62
Rotation speed	2	6.91	26.8
Abrasive concentration	2	2.08	8.1
Size of abrasive particle	2	0.79	3.1
Total	8	25.74	100

**Table 9:** Experimental conditions.

Process Parameters	DEPP Method	CMP Method
Workpiece	3-inch silicon wafer	3-inch silicon wafer
Abrasive particle	SiO_2_	SiO_2_
Size of abrasive particle (nm)	80	80
Abrasive concentration (wt%)	30	30
pH of polishing slurry	9.5	9.5
Rotation speed (rpm)	90	90
Pressure (kPa)	8.38	8.38
Electric field intensity (V/mm)	450	–
Frequency (Hz)	40	–
